# Phantom Vibration Syndrome and Problematic Smartphone Use Among University Students: Associations With Sleep Disturbance, Mental Health, and Academic Stress

**DOI:** 10.7759/cureus.103567

**Published:** 2026-02-13

**Authors:** Maherin Khan, Mayren Heshmat Abdelalim Abdalla Mansour, Ashish Bishnoi, Priyanshu Dixit, Victor C Onuabuchi, Sepher K Khiavi, Hadiya Aleem, Ayaz Ali, Prachi Kumari, Hansi Zhang

**Affiliations:** 1 College of Basic Medical Sciences, Jilin University, Changchun, CHN; 2 Public Health, Jilin University, Changchun, CHN; 3 Public Health Dentistry, Jilin University, Changchun, CHN; 4 Biochemistry, University of Delhi, Delhi, IND

**Keywords:** anxiety, mental health, phantom vibration syndrome, problematic smartphone use, sleep disturbances

## Abstract

Background: In a world where the mind vibrates even when the phone does not, digital habits are quietly reshaping how we rest, focus, and feel. Phantom vibration syndrome (PVS), the false sensation of a phone vibrating, and problematic smartphone use (PSU) have become increasingly common among university students. These behaviors may reflect deeper psychological conditioning linked to anxiety, poor sleep, and stress, yet they remain underexplored from a public health perspective.

Methods: A cross-sectional online survey was conducted among 553 university students to examine the prevalence and correlates of PVS and PSU. Standardized scales measured sleep disturbance, anxiety symptoms, and academic stress. Descriptive and inferential analyses were performed using IBM SPSS Statistics version 29.0 (IBM Corp., Armonk, NY, USA). An open-ended question invited students to share strategies for managing smartphone use, and responses were analyzed thematically to identify recurring digital well-being themes.

Results: Phantom vibrations were reported by 41.4% of participants, with 22% experiencing them occasionally and 5.4% frequently. More than half (54.8%) reported fatigue and poor sleep. Higher PSU scores were significantly associated with anxiety (p<0.01) and sleep disturbance (p<0.05). Qualitative insights revealed three consistent coping patterns: digital detox routines, environmental changes, and structured daily activities that limited device use.

Conclusions: The findings suggest that the modern student's constant connectivity has subtle but measurable effects on mental and physical well-being. By treating digital balance as an essential health behavior, similar to sleep hygiene or nutrition, universities can help protect students from the psychological fatigue of always being online. Addressing PVS and PSU through awareness and behavioral interventions should be considered a public health priority for the digital age.

## Introduction

It is a silent expectation that never materialized but has a strong mental resonance. This brief feeling has become a commonplace aspect of digital life for the younger generation [1], serving as a subtly potent reminder of how deeply technology has permeated human behavior. Reaching for a phone that never vibrated or checking a screen that remained dark, once an occasional distraction, has become an almost universal reflex.

Excessive smartphone use has rapidly evolved into a modern behavioral epidemic, affecting not just productivity but the very rhythms of sleep, mood, and mental focus among young adults [1,2]. Beyond mere screen exposure, specific behaviors such as phantom vibration syndrome (PVS) and problematic smartphone use (PSU) reflect psychological dependency created by constant digital connectivity [3-5].

While recent research has explored the consequences of excessive smartphone exposure, much of it focuses narrowly on screen time or social media addiction, overlooking subtler phenomena such as PVS [6,7]. Similarly, although PSU has been linked to heightened anxiety, poor sleep, and academic strain, studies often rely on small or homogeneous samples [8,9]. A substantial knowledge gap persists in understanding how these interconnected digital behaviors jointly affect mental and physical well-being across culturally diverse academic populations [10-12].

University students represent a particularly vulnerable demographic, caught between academic demands, social pressures, and the constant pull of technology [13,14]. The convergence of these stressors fosters an environment where compulsive smartphone engagement becomes normalized, and its health implications are overlooked.

This study, therefore, investigates the combined impact of PVS and PSU on sleep disturbance, anxiety, and academic stress among university students. By examining these emerging digital behaviors together, this research seeks to illuminate their collective contribution to the growing public health crisis of digital fatigue. Addressing this silent epidemic is crucial: just as diet, sleep, and physical activity define wellness, digital well-being must now be recognized as a core determinant of health in the 21st century.

This article was previously posted as a preprint on the Research Square platform [15].

Objectives

The primary objective of this study was to examine the prevalence of PVS among university students and to assess its associations with PSU, self-reported sleep disturbances, and mental health-related symptoms. Secondary objectives included exploring patterns of smartphone-related behaviors, such as nighttime phone checking, and qualitatively summarizing students’ reported coping strategies related to digital well-being.

## Materials and methods

Study design

This cross-sectional study examined associations between PVS, PSU, self-reported sleep disturbances, and mental health-related symptoms among university students. Data were collected via a self-administered online questionnaire distributed between April and May 2025. The survey included structured and open-ended items to assess smartphone-related behaviors, emotional reliance, and psychological symptoms experienced over the previous two weeks.

Study population

**Figure 1:**
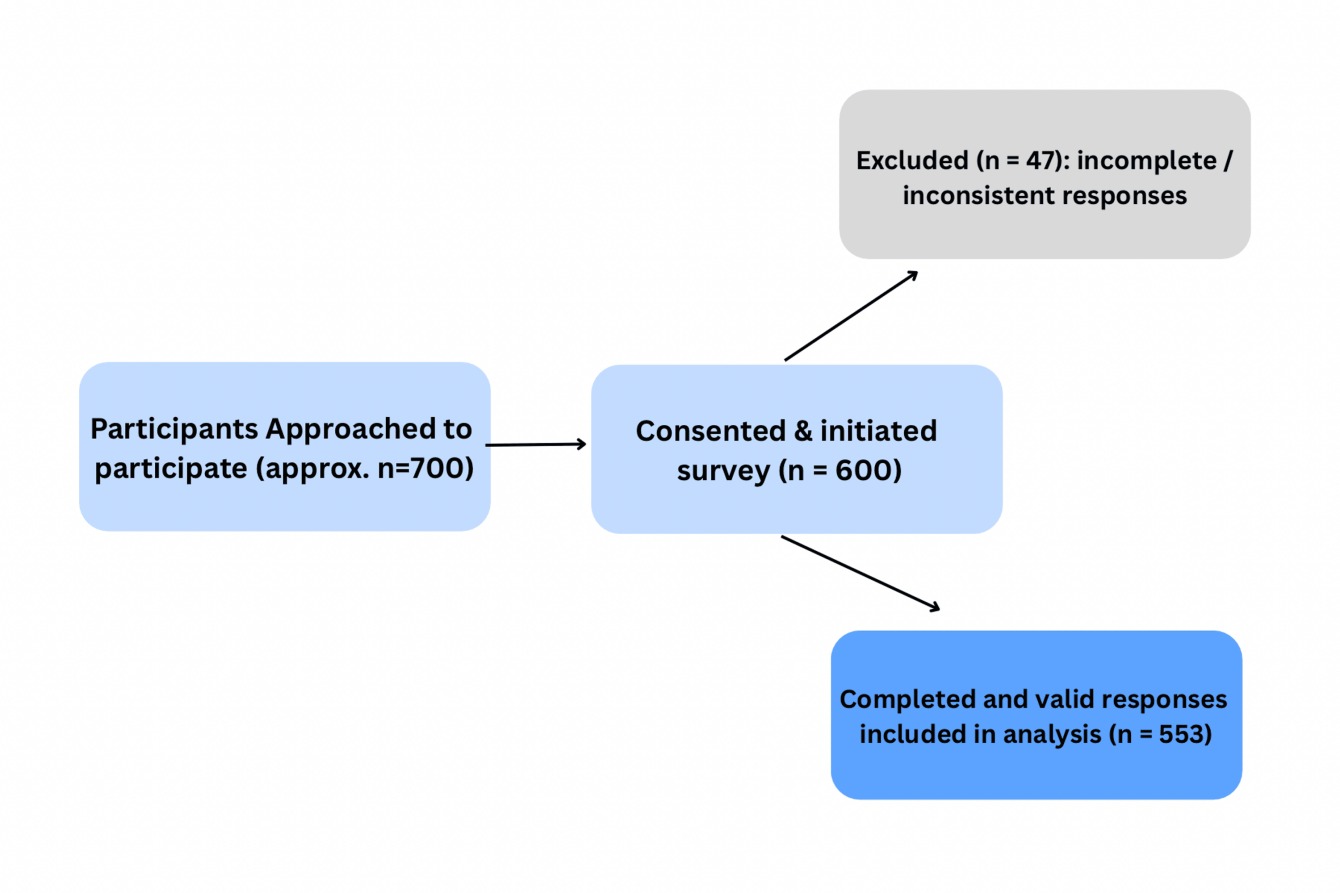
Participant recruitment and inclusion flow diagram

Undergraduate and postgraduate students enrolled in various academic programs at Jilin University were eligible if they were currently enrolled, aged 17 years or older, and provided informed consent. A non-probability convenience sampling method was used. Survey posters with QR codes were circulated physically and electronically to maximize outreach. Approximately 700 students were reached, 600 initiated the survey, and 553 provided complete and valid responses for analysis. Incomplete or inconsistent responses were excluded. A flow diagram illustrating participant recruitment and selection is presented in Figure 1.

Data collection and measures

The questionnaire consisted of five domains: sociodemographic characteristics, PVS, smartphone use behaviors, self-reported sleep disturbances, and recent mental health-related symptoms.

Phantom Vibration Syndrome

This was assessed based on the presence, frequency, and situational context of perceived phantom vibrations.

Sleep Disturbances

Participants self-reported sleep quality and sleep-related complaints, including fatigue and perceived sleep disruption, rather than clinically diagnosed sleep disorders.

Mental Health-Related Symptoms

These were assessed using selected items adapted from the Patient Health Questionnaire-9 (PHQ-9) [16] and Generalized Anxiety Disorder-7 (GAD-7) [17] to screen for fatigue, low mood, worry, and anxiety. Selected items were used for symptom screening purposes only; full diagnostic scales were not administered, and no clinical diagnoses were intended.

Smartphone Use Behaviors

These included daily screen time, emotional attachment to smartphones, and use during pre-sleep or nighttime hours [18].

Open-Ended Question

Participants were asked to suggest one change that could improve overall well-being or digital habits. Responses were reviewed and thematically grouped to identify recurring patterns related to digital well-being strategies.

Statistical analysis

Data were entered into Microsoft Excel and analyzed using IBM SPSS Statistics Version 29.0 (IBM Corp., Armonk, NY, USA).

Descriptive statistics were calculated for all variables. Categorical variables were summarized using frequencies and percentages, whereas continuous variables were reported as means and standard deviations.

Bivariate associations between PVS and categorical variables, including daily screen time, nighttime phone checking, sleep quality, and anxiety-related symptoms, were examined using chi-square (χ²) tests. A p-value <0.05 was considered statistically significant.

Binary logistic regression models were conducted to assess the relationship between PVS and key health outcomes. Two separate models were constructed: Model 1 assessed anxiety symptoms (present vs. absent), and Model 2 assessed poor sleep quality (poor/very poor vs. good/very good).

PVS status (yes vs. no) was the primary independent variable. Models were adjusted for potential covariates, including age group, gender, and daily screen time (>6 hours vs. ≤6 hours). Results were reported as odds ratios (ORs) with 95% CI and corresponding p-values. All statistical tests were two-tailed. The full questionnaire used in the study is provided in Table 1. Responses with substantial missing data or internally inconsistent answers were excluded before analysis.

**Table 1:** Study questionnaire

Item no.	Questionnaire item	Response options
1	Age (in years)	Numeric
2	Gender	Male / female / prefer not to say
3	Academic level	Undergraduate / postgraduate
4	Field of study	Open-ended
5	Have you ever experienced a sensation where you felt your phone vibrating when it was not?	Yes / no
6	Frequency of phantom vibration sensations	Rarely / occasionally / frequently
7	Situations in which phantom vibrations occur	Study / rest / stress / night / other
8	Average daily smartphone screen time	<2 h / 2-4 h / 4-6 h / >6 h
9	Emotional attachment to smartphone	Not at all / slightly / moderately / strongly
10	Smartphone use before sleep	Yes / no
11	Wake up at night to check phone	Never / occasionally / frequently
12	Overall sleep quality	Very good / good / poor / very poor
13	Daytime fatigue or sleepiness	Yes / no
14	Smartphone use affects sleep	Yes / no
15	Feeling nervous or anxious (past 2 weeks)	Not at all → nearly every day
16	Difficulty relaxing	Not at all → nearly every day
17	Feeling tired or low energy	Not at all → nearly every day
18	Feeling down or low mood	Not at all → nearly every day
19	Suggested change to improve digital habits	Open-ended

Open-ended responses were grouped thematically using an exploratory descriptive approach rather than formal qualitative coding procedures.

Ethical approval

This study was conducted in accordance with the Declaration of Helsinki [19]. Ethical approval was obtained from the Institutional Review Board of Jilin University (AF-BMSIRB-01-07). All participants were informed about the study’s purpose and assured of anonymity and confidentiality.

The questionnaire was developed after reviewing relevant literature on PVS, PSU, sleep-related complaints, and mental health symptoms among university students. Items were designed to capture self-reported behaviors and symptoms rather than clinical diagnoses. Selected screening items were adapted from previously validated instruments, including the PHQ-9 and GAD-7, to assess recent mental health-related symptoms. The questionnaire was informally pretested among a small group of students to ensure clarity and comprehensibility before final distribution. Only selected screening items were adapted from the PHQ-9 and GAD-7 instruments; the full scales were not administered, and no diagnostic interpretations were made. This approach was adopted to minimize respondent burden and improve survey completion, and the selected items were analyzed individually as symptom indicators rather than as psychometric scale measures. Because the full PHQ-9 and GAD-7 instruments were not administered and the items were not intended to form composite scales, internal consistency measures such as Cronbach’s alpha were not calculated.

## Results

Participants characteristics

A total of 553 students participated in the study. The majority were aged 17-23 years (78.7%) and identified as East Asian (79.4%). Gender distribution was balanced, and over half studied ≥4 hours per day with attendance rates above 90% (Table 2).

**Table 2:** Sociodemographic characteristics of the study participants

Characteristics	Category	n (%)
Age group	17-23 years	435 (78.7%)
	24-27 years	81 (14.6%)
	≥28 years	37 (6.7%)
Gender	Male	291 (52.6%)
	Female	262 (47.4%)
Ethnicity	East Asian	244 (44.1%)
	Middle Eastern	109 (19.7%)
	South Asian	106 (19.2%)
	African	56 (10.1%)
	European	38 (6.87%)
Year of study	1st year	167 (30.2%)
	2nd year	119 (21.5%)
	3rd year	163 (29.5%)
	4th year	74 (13.4%)
	5th year	30 (5.4%)
Daily study hours	<2 hours	60 (10.8%)
	2-4 hours	149 (26.9%)
	4-6 hours	178 (32.2%)
	>6 hours	166 (30.0%)
Attendance rate	>90%	386 (69.8%)
	80-89%	92 (16.6%)
	60-79%	38 (6.9%)
	<60%	37 (6.7%)
Daily screen time	<2 hours	23 (4.2%)
	2-4 hours	102 (18.4%)
	4-6 hours	187 (33.8%)
	6-8 hours	121 (21.9%)
	>8 hours	120 (21.7%)

Prevalence and characteristics of phantom vibration syndrome

**Figure 2:**
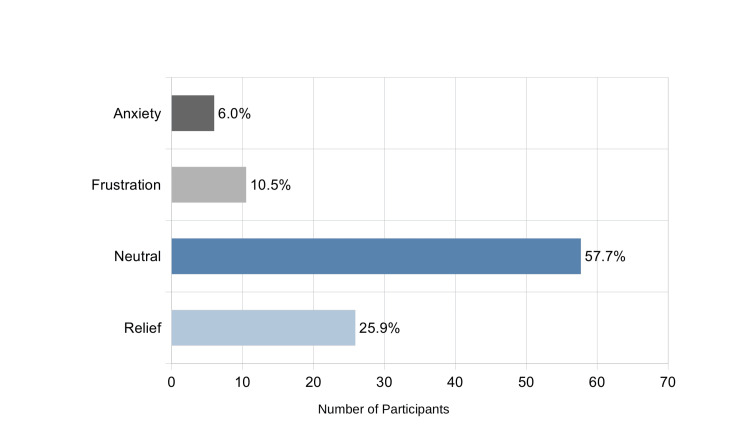
Emotional responses to phantom vibration experiences among students reporting PVS (n=229) PVS, phantom vibration syndrome

Overall, 41.4% (n=229) of respondents reported experiencing phantom vibrations. Among these, most reported rare episodes (62.6%), while 32.0% experienced them occasionally and 5.4% frequently. PVS episodes occurred most often while studying (48.1%) and during class (33.8%), followed by while communicating (27.5%) or before sleep (22.8%). Emotional responses to PVS were largely neutral (57.7%), but 25.9% reported relief, 10.5% frustration, and 6.0% anxiety (Figure 2).

Smartphone use behaviors

**Figure 3:**
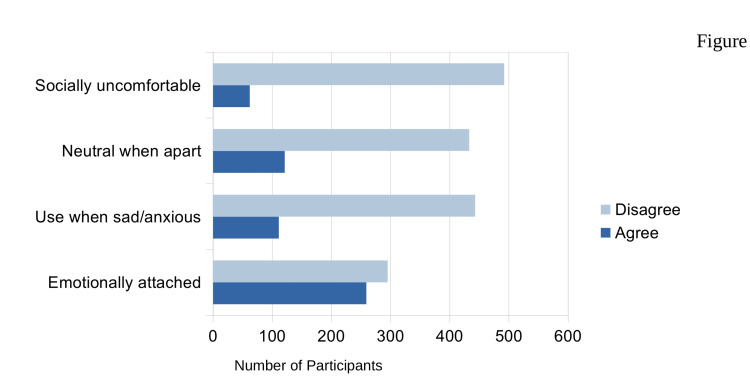
Emotional and social patterns of smartphone use among university students (n=553)

Most participants reported using their smartphones within 30 minutes before bedtime, and over half spent more than four hours daily on their devices. Academic performance and sleep schedules were the most commonly affected domains, while social interaction was less frequently reported. Emotional attachment to smartphones was widespread, with many participants describing affective dependence on their devices (Figure 3).

Sleep quality and mental health-related symptoms

Overall, 22.4% of students reported poor or very poor sleep quality. Fatigue and low energy were the most commonly reported symptoms (54.8%), followed by anxiety-related symptoms such as trouble relaxing (42.9%) and excessive worry (40.7%). Table 3 summarizes the prevalence of sleep and mental health indicators in the study population.

**Table 3:** Self-reported sleep quality and mental health-related symptoms among university students (N=553) Selected items were adapted for symptom screening and were not administered in full. PHQ-9, Patient Health Questionnaire-9; GAD-7, Generalized Anxiety Disorder-7

Variable	n	%
Sleep quality		
Good / very good	429	77.6
Bad / very bad	124	22.4
Mental health-related symptoms		
Fatigue / low energy	303	54.8
Sleep troubles	192	34.7
Loss of interest	200	36.2
Depressive symptoms / hopelessness	159	28.8
Concentration issues	163	29.5
Trouble relaxing (anxiety-related)	237	42.9
Nervous / anxious feelings	225	40.7
Excessive worry	225	40.7
Irritability	132	23.9

Associations between phantom vibration syndrome, smartphone use, and well-being

Chi-square analyses revealed significant associations between PVS experience and several behavioral and mental health factors. Students who experienced PVS were more likely to report high daily screen time (>6 hours; χ²(1)=12.4, p=0.002) and nighttime phone checking (χ²(1)=9.1, p=0.01) compared to those without PVS. PVS was also significantly associated with anxiety symptoms (χ²(1)=15.6, p<0.001). No significant associations were found with gender or academic year.

To further examine factors independently associated with PVS, a multivariable binary logistic regression analysis was performed. After adjustment for gender and daily screen time, anxiety-related symptoms remained significantly associated with PVS (OR=1.51, 95% CI: 1.07-2.14, p=0.019), indicating higher odds of PVS among students reporting anxiety symptoms. Female gender and high daily screen time were not independently associated with PVS in the adjusted model (p>0.05).

Qualitative responses

**Figure 4:**
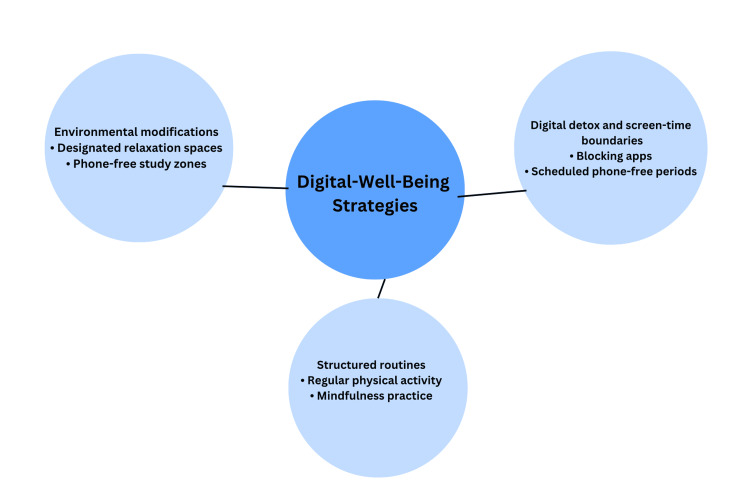
Thematic map summarizing digital well-being strategies identified by students. Key approaches included structured digital detox periods, mindful environmental design, and routine-based behavioral regulation

Thematic analysis of 112 open-ended responses identified three dominant strategies proposed by students to reduce stress and improve well-being: (1) digital detox and screen-time boundaries, such as using blocking apps or scheduling phone-free periods; (2) environmental modifications, including designated relaxation spaces at university; and (3) structured routines, particularly integrating regular physical activity and mindfulness practices. A thematic map (Figure 4) was developed based on the themes identified through this analysis [20].

## Discussion

The present study investigated associations between PVS, PSU, self-reported sleep disturbances, and mental health-related symptoms among university students. Overall, our findings indicate that students experiencing PVS reported higher daily smartphone use, more frequent nighttime checking, and greater self-reported anxiety- and depression-related symptoms. Fatigue and low energy were the most commonly reported symptoms, consistent with prior research on the psychological impact of excessive smartphone engagement.

The observed relationships may be interpreted in light of conditioning theory and the fear of missing out (FOMO), although these interpretations remain hypothetical and should be explored further using longitudinal or experimental designs [21]. Given the cross-sectional nature of this study, causal relationships between PVS, PSU, self-reported sleep disturbances, and mental health-related symptoms cannot be inferred. It is also plausible that self-reported sleep disturbances may precede or exacerbate PVS and PSU, as poor sleep quality can increase cognitive vigilance and emotional sensitivity, potentially heightening the perception of phantom sensations.

From a behavioral perspective, repeated exposure to smartphone notifications and habitual checking behaviors may reinforce heightened sensory vigilance, which could plausibly contribute to the perception of phantom vibrations among frequent users. In the present study, the higher prevalence of PVS among students reporting increased screen time, nighttime checking, and anxiety-related symptoms is consistent with this conceptual framework. However, these interpretations remain exploratory and should be evaluated in future longitudinal studies.

**Figure 5:**
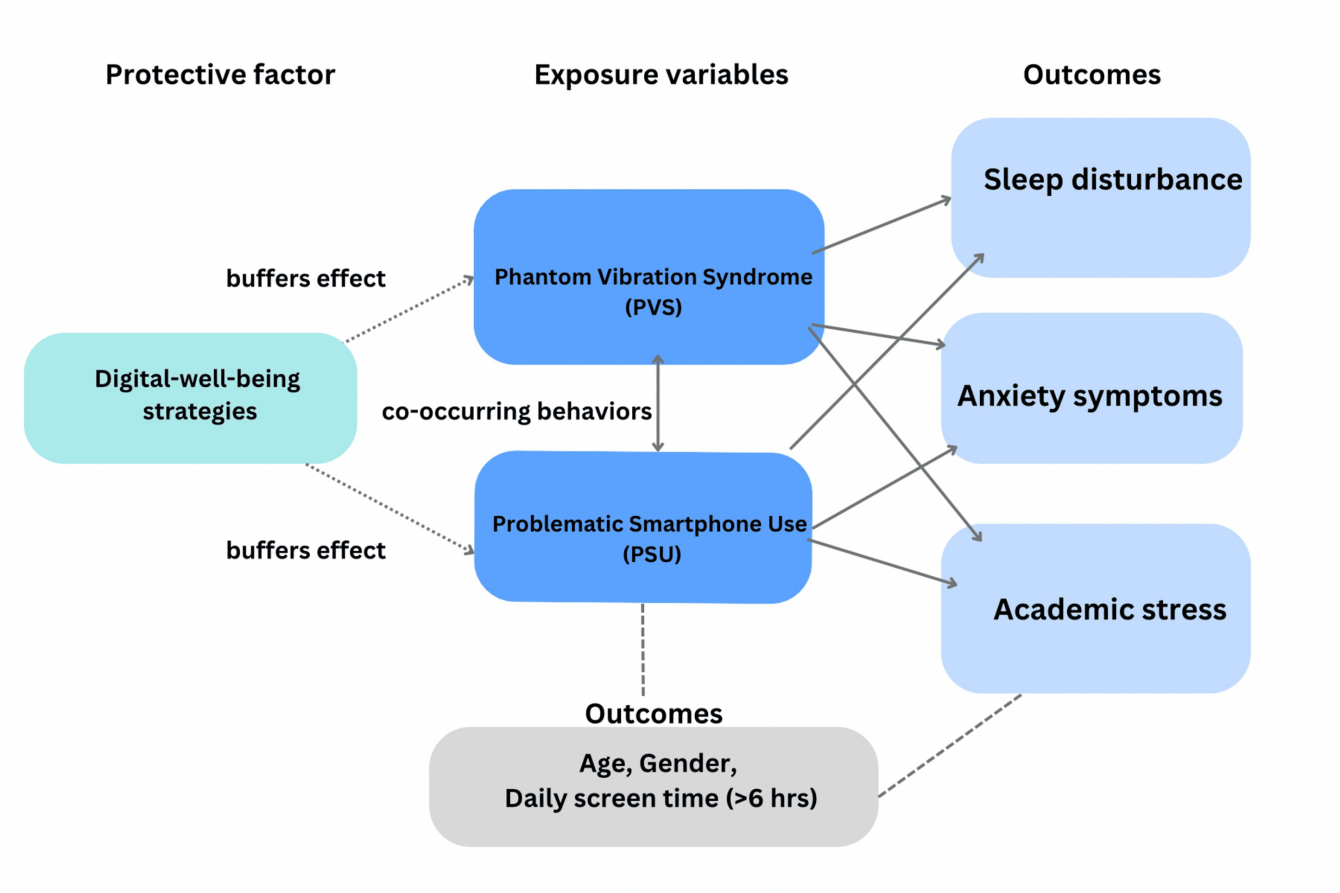
Conceptual model illustrating hypothesized pathways between PVS, PSU, and mental health outcomes. PVS and PSU are proposed to influence sleep disturbance, anxiety symptoms, and academic stress. Digital well-being strategies may buffer these effects, while age, gender, and daily screen time (>6h) are considered potential confounders PVS, phantom vibration syndrome; PSU, problematic smartphone use

The patterns identified in this study align with international evidence. Studies in China, Norway, and Turkey have likewise reported associations between prolonged smartphone use and symptoms of anxiety and sleep disturbance [2,7,9]. However, our findings extend these observations to a culturally diverse academic cohort, demonstrating that digital dependency transcends geographic boundaries. The emotional attachment and pre-sleep usage habits observed in this study mirror global behavioral trends, reinforcing that excessive smartphone engagement is an emerging public health concern with broad relevance [22]. The observed trends in PVS in relation to smartphone use and psychological factors are summarized in Figure 5.

Nighttime smartphone use may additionally influence sleep and psychological well-being through behavioral and perceptual mechanisms [23]. Participants in this study frequently reported nighttime phone checking and perceived sleep disruption, which may contribute to fatigue and emotional strain. These subjective experiences may help explain the observed associations between excessive smartphone use, self-reported sleep disturbances, and mental health-related symptoms, without implying specific physiological or clinical pathways.

Our study highlights the importance of considering both behavioral and emotional aspects of smartphone engagement when evaluating digital well-being. Emotional attachment to smartphones, nighttime checking behaviors, and daily screen time were all associated with self-reported mental health-related symptoms and sleep disturbances. These findings underscore the potential value of preventive strategies aimed at promoting balanced smartphone use and improved digital habits among university students [24].

Several limitations should be noted. The use of convenience sampling and a single university limits generalizability. Sleep quality and mental health-related symptoms were assessed using self-reported measures and selected items from validated screening tools rather than full diagnostic instruments, which limits comparability with studies using complete questionnaires. The cross-sectional design prevents the determination of causality, and academic program-specific analyses were not conducted, which may have revealed differential patterns across disciplines. Despite these limitations, the study included a relatively large and homogeneous sample, allowing for meaningful evaluation of associations among PVS, PSU, self-reported sleep disturbances, and mental health-related symptoms. Due to the nature of the study, the directionality of these associations cannot be determined, and reciprocal relationships between smartphone use, sleep disturbance, and psychological symptoms are possible.

## Conclusions

This study highlights that PVS and PSU are prevalent and interconnected behaviors among university students, with measurable associations with anxiety symptoms, poor sleep quality, and academic stress. These patterns illustrate how constant digital engagement can heighten psychological arousal, disrupt rest, and undermine overall well-being.

Beyond identifying risk factors, the qualitative findings revealed that students themselves recognize the importance of behavioral change. Many proposed simple, self-directed strategies such as digital detox periods, structured daily routines, physical exercise, and designated relaxation spaces on campus. These student-driven suggestions reflect a growing awareness of the psychological costs of excessive connectivity and point toward practical, low-cost solutions that can be implemented within academic settings. At a broader level, the findings underscore the need for universities and public health stakeholders to adopt integrated digital well-being strategies that promote balanced smartphone use, sleep hygiene, and mental health awareness. Introducing supportive environments such as phone-free study zones, wellness workshops, and spaces for relaxation could help students manage stress and enhance focus.
